# Computational prediction of changes in brain metabolic fluxes during Parkinson’s disease from mRNA expression

**DOI:** 10.1371/journal.pone.0203687

**Published:** 2018-09-12

**Authors:** Farahaniza Supandi, Johannes H. G. M. van Beek

**Affiliations:** 1 Department of Clinical Genetics, VU University Medical Centre, Amsterdam, the Netherlands; 2 Institute of Biological Sciences, Faculty of Science, University of Malaya, Kuala Lumpur, Malaysia; 3 Department of Experimental Vascular Medicine, Academic Medical Center, AZ Amsterdam, the Netherlands; Mayo Clinic Rochester, UNITED STATES

## Abstract

**Background:**

Parkinson’s disease is a widespread neurodegenerative disorder which affects brain metabolism. Although changes in gene expression during disease are often measured, it is difficult to predict metabolic fluxes from gene expression data. Here we explore the hypothesis that changes in gene expression for enzymes tend to parallel flux changes in biochemical reaction pathways in the brain metabolic network. This hypothesis is the basis of a computational method to predict metabolic flux changes from post-mortem gene expression measurements in Parkinson’s disease (PD) brain.

**Results:**

We use a network model of central metabolism and optimize the correspondence between relative changes in fluxes and in gene expression. To this end we apply the Least-squares with Equalities and Inequalities algorithm integrated with Flux Balance Analysis (Lsei-FBA). We predict for PD (1) decreases in glycolytic rate and oxygen consumption and an increase in lactate production in brain cortex that correspond with measurements (2) relative flux decreases in ATP synthesis, in the malate-aspartate shuttle and midway in the TCA cycle that are substantially larger than relative changes in glucose uptake in the substantia nigra, dopaminergic neurons and most other brain regions (3) shifts in redox shuttles between cytosol and mitochondria (4) in contrast to Alzheimer’s disease: little activation of the gamma-aminobutyric acid shunt pathway in compensation for decreased alpha-ketoglutarate dehydrogenase activity (5) in the globus pallidus internus, metabolic fluxes are increased, reflecting increased functional activity.

**Conclusion:**

Our method predicts metabolic changes from gene expression data that correspond in direction and order of magnitude with presently available experimental observations during Parkinson’s disease, indicating that the hypothesis may be useful for some biochemical pathways. Lsei-FBA generates predictions of flux distributions in neurons and small brain regions for which accurate metabolic flux measurements are not yet possible.

## Introduction

Many human diseases are associated with changes in metabolism at the cellular level. Metabolic fluxes are hard to measure in patients, but changes in expression of metabolic genes during disease are often measured, with spatial resolution down to the level of small anatomical regions and even specific cell types. It would therefore be of interest to predict changes in metabolic fluxes from gene expression measurements during disease.

It has long been considered difficult to predict changes in metabolic fluxes from the gene expression changes [1]. Nevertheless, at least eighteen algorithms exist to infer large models of metabolism and predict metabolic flux distribution from gene expression [2]. Seven of the most used algorithms have been tested in yeast, comparing metabolic flux predictions based on gene expression with measurements of intracellular and extracellular fluxes based on ^13^C labeling data, but the algorithmic predictions turned out to be of low quality and were in several cases worse than predictions by parsimonious Flux Balance Analysis which does not even take gene expression into account [2]. We applied the algorithms tested by Machado and Herrgard [2] to derive metabolic fluxes in Alzheimer’s disease from gene expression, but the metabolic predictions did not agree with measurements of oxygen and glucose uptake rates [3]. Many of the most used algorithms from Machado’s test suite, e.g. iMAT [4], rely on complete inactivation of reactions that are linked to genes with low expression. However, gene expression measurements during Parkinson’s disease show relatively small changes in expression of many genes related to metabolism. Such modest changes are not compatible with complete inactivation of biochemical reactions in the model analysis. Here we therefore explore a recently published algorithm [3], Lsei-FBA, that does not rely on complete inactivation of reactions and might be suitable to predict changes in metabolism from measured small changes in gene expression across a metabolic network. The Lsei-FBA algorithm is based on the hypothesis that relative changes in reaction fluxes in biochemical pathways parallel changes in gene expression at the level of metabolic networks. This hypothesis does not mean that changes at the level of a single biochemical reaction are also proportional to expression levels of the enzyme or the gene expression of the associated gene(s). The hypothesis has been discussed extensively in [3]. The hypothesis will be tested in the present study by comparing predictions of the Lsei-FBA algorithm with available experimental data.

Parkinson’s disease (PD) is one of the most widespread neurodegenerative disorders. PD is characterized among others by movement disorder, rigidity and tremor caused by the loss of dopaminergic neurons in the substantia nigra pars compacta (SNc) of the brain. Although several genes have been identified in familial cases and by genome wide association analysis, the mechanisms for the PD progression are largely unknown. Damage to the mitochondria resulting in failure to generate energy possibly contributes to PD [5,6]. Several gene products linked to PD show mitochondrial localizations. Mitochondrial dysfunction has also been implicated in other neurodegenerative diseases such as Alzheimer’s disease (AD), Huntington’s disease (HD) and Amyotrophic Lateral Sclerosis (ALS) [7].

PD is in particular often associated with disturbed mitochondrial function in the neurons in the SNc which are the most conspicuous target of the disease. Decrease in complex I activity in the electron transport chain (ETC) during PD has been measured in the substantia nigra [8] and frontal cortex [9] of post-mortem brain. Reduction of other ETC complexes (II, III and IV) has been reported for the substantia nigra, platelets and muscle (reviewed in [5,10]). Statistical analysis of gene expression also suggests that mitochondrial electron transport and glucose metabolism in the SNc and other brain regions are affected [11]. However, the pattern and the magnitude of the changes in metabolic flux distribution are unknown. Accurate measurements of metabolic fluxes in the small brain regions and cell types targeted by PD are presently impossible. Measurements of oxygen and glucose uptake with positron emission tomography (PET) in PD patients have been done for larger brain regions [12], and increased lactate accumulation has been measured with NMR spectroscopy [13]. Because it is difficult to measure metabolic reaction rates directly in small brain regions or in specific cell types, it is useful to predict redistribution of metabolism from mRNA expression measured in the small regions affected by PD, such as the SNc and specifically in dopaminergic neurons.

In the present study we report quantitative predictions of the changes during PD in the distribution of fluxes in central energy metabolism in specific small brain regions and in dopaminergic neurons. The first aim of the study is to explore whether the recently developed Lsei-FBA algorithm can predict changes in metabolism from measured changes in gene expression during human disease that agree with experimental data in direction and approximate magnitude of change. In case this works well for the available data, the hypothesis of parallel changes in gene expression and flux in biochemical pathways appears to be useful, at least for some biochemical pathways, and the algorithm’s predictions gain confidence. Consequently, the second aim of the study can then be to predict the complete change in flux distribution in the central metabolic network, containing 69 biochemical reactions, from gene expression measurements in small brain regions during PD.

## Materials and methods

### Metabolic model reconstruction for brain metabolism

A metabolic reaction network was constructed consisting of the major pathways representing central carbon and energy metabolism in the brain. Rather than relying on a genome-wide metabolic reconstruction of brain metabolism, we chose a manually curated representation of central metabolism. The detailed rationale for this model and an extensive comparison with a larger model of brain metabolism has been described in [3]. Metabolites and enzymatic reactions were distributed over the extracellular, cytosolic and mitochondrial compartments. The pathways include glycolysis, pentose phosphate pathway (PPP), TCA cycle, oxidative phosphorylation (OxPhos), reducing equivalent shuttling mechanisms, gamma-aminobutyric acid (GABA) shunt and transport of metabolites across the membranes which separate the compartments. We updated this model by adding the glutamate-glutamine cycle, pyruvate carboxylase reaction and ammonium transport across the mitochondrial membrane. The selected reactions were imported from the BiGG database [14]. Complete lists of the reactions in the network along with the lists of metabolites are given in S1 and S2 Tables. S1 Fig shows a scheme of the network.

### Analysis of mRNA expression data

Datasets containing the CEL files with gene expression data of individual post-mortem brain samples for neuropathologically confirmed PD patients and normal controls from the same study were downloaded from the Gene Expression Omnibus (GEO) database [15] and the National Brain Databank (NBD; http://national_databank.mclean.harvard.edu/brainbank/Main) and are summarized in S3 Table. The datasets are given in [11,16–22]. The dataset from Cantuti-Castelvetri *et al*. [23] (GEO accession GSE24378) was excluded from the flux analysis presented in this paper for reasons given in the Discussion; however the result of the Lsei-FBA analysis of this study is still given in S4 Table.

All Affymetrix CEL files were pre-processed and normalized in the R programming environment using the RMA method [24]. Log2 transformed values were used to calculate differences in expression levels of PD patients against the healthy controls.

### Mapping of expression data on a pathway map

Based on the reactions in our network, a visual map was drawn incorporating pathways downloaded from KEGG [25] and WikiPathways [26] and modified manually in the pathway visualization tool PathVisio [27]. Log2 transformed gene expression data were mapped on the metabolic model using the visualization options of the PathVisio tool, see S2 Fig.

### Analysis of flux distribution: The Lsei-FBA algorithm

Recently it was reported that metabolic fluxes in yeast can be meaningfully predicted based on absolute gene expression in yeast [28]. Here we apply an approach to predict *changes* in metabolic flux distribution during disease from *changes* in gene expression in human tissue. Our approach, termed Lsei-FBA, was recently described and demonstrated on a data set for Alzheimer’s disease [3]. Lsei-FBA is based on the hypothesis that on average relative changes in flux in a biochemical pathway are proportional to changes in gene expression of the associated with the enzymes in that pathway. The hypothesis is to be applied on the metabolic network level, and the parallel changes are not assumed to hold at the level of individual enzymes and their associated reactions. Lsei-FBA does not provide an exact calculation based on enzyme kinetic equations, enzyme activities and metabolite concentrations, but a bioinformatic prediction of changes at the network level based on the tendencies suggested by gene expression changes. It builds on the idea which is widely used in genomics that it is possible to predict changed activity of biological pathways from associated gene expression changes. Rather than testing statistical significance of changes in expression for a group of genes associated with a particular biological pathway, Lsei-FBA projects the changes in gene expression on a model of the connected metabolic network from which changes in flux distribution in the network as an integrated whole are predicted. The Lsei-FBA approach is applied here to predict metabolic changes during Parkinson’s disease from gene expression data.

**Fig 1:**
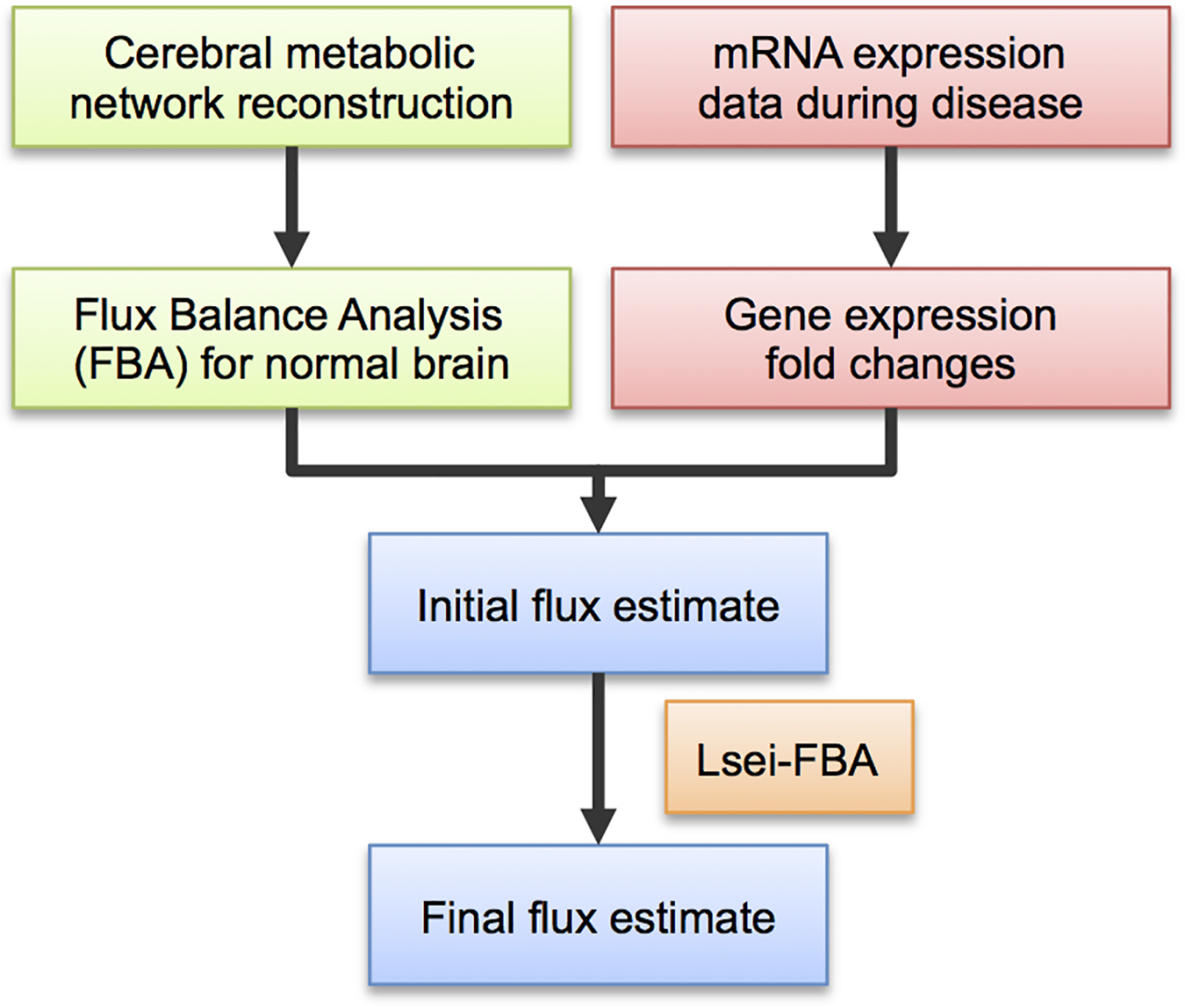
Diagram for the workflow of the Lsei-FBA approach. Flow diagram of the steps to predict metabolic fluxes for the normal brain (green boxes) and for diseased brain based on gene expression data (pink boxes) described in the Methods section. For the normal brain, the flux distribution was computed from a reconstructed model of cerebral central carbon metabolism. For the diseased brain, mRNA gene expression fold changes were first computed for patients with Parkinson’s disease (PD) versus a control group. An initial flux estimate for the diseased brain is computed for each reaction in the network by multiplying gene expression fold changes with the FBA flux predictions for the normal brain. The final flux estimate is solved subject to i) forward flux in irreversible reactions, ii) maintaining the balance of fluxes during chronic disease and iii) a least squares cost function to minimize the sum of the squared deviations between the initial and the final flux estimate.

The Lsei-FBA approach to predict changes in central energy metabolism during PD starts with establishing the metabolic flux distribution in the normal brain based on measured data for the uptake and production of metabolites in healthy human brain [3]. This data is analyzed using flux balance analysis (FBA) of a network model of central energy metabolism to predict the flux distribution in normal brain. The change in flux distribution during PD is then calculated based on our assumption that, on average, the flux carried by each enzyme tends to change proportionally to the change of its mRNA expression between controls and PD patients. Note that we do not assume that every reaction rate changes in proportion to the gene expression level, but that on average the reaction fluxes tend to follow gene expression. The steps describing the method are summarized in Fig 1.

We maintain the assumption of balance of fluxes in the metabolic network also for the diseased state, because metabolites that are not exchanged between brain tissue and blood cannot keep accumulating steadily during chronic disease, and their production and consumption must therefore be approximately balanced. The changes in mRNA expression provide a first rough prediction of the change in metabolic fluxes based on the assumption that the relative change in gene expression and in metabolic flux for the genes tend to correspond. Consequently, the initial rough estimate for the flux in each reaction is: flux in the healthy state for that reaction times the fold change in associated gene expression. This initial set of estimates is refined by using the consistency and balance of fluxes in the metabolic network as additional constraints. The assumption of proportionality between gene expression and enzymatic flux, at least on average, will be discussed below.

The final prediction of metabolic fluxes is subject to 1) flux balance in the network for metabolites which are not exchanged between brain and blood 2) restriction to forward flux through irreversible reactions 3) maximization of correspondence between relative changes in mRNA expression and changes in fluxes. We include expression datasets from the SNc and from laser captured microdissected (LCM) dopaminergic neurons where neuronal damage usually occurs most prominently during PD. These SNc measurements are compared with other brain regions that show abundant Lewy bodies (LB) in PD without neuronal loss, such as frontal cortex, prefrontal cortex Brodmann area 9 (BA9) and basal ganglia structures. A statistical meta-analysis at the gene-set level of these datasets [11] was already reported, showing significant changes in mitochondrial electron transport and glucose metabolism, and is not repeated here.

### Flux balance analysis for the healthy brain

A list of reaction equations was prepared according to the reaction list in the BiGG database (S1 Table). The metabolic system is assumed to be in steady state. Substrate uptake measurements for the healthy elderly (55–65 years) human brain were taken from [29], which reported the uptake rates of glucose, and release of lactate, glutamine and pyruvate for the brain to be 0.203, -0.0092, -0.011 and -0.0024 μmol g wet brain^-1^ min^-1^, respectively. A small flux is measured in the PPP in the normal brain, which amounts to 6.9% of glycolysis [30]. Pyruvate carboxylation and glutamate-glutamine cycling fluxes amount to 13% and 62% of the value of the total glucose uptake in the brain, respectively [31] while the GABA shunt flux is 32% of the glucose uptake value [32]. These exchange rates and relative flux values were used as constraints in the model.

Flux balance analysis for the normal brain was done assuming a cost function which maximizes ATP synthesis. The rationale for this assumption was discussed extensively in [3]. Assuming maximal growth, which is often used for flux balance analysis of bacterial metabolism, is inappropriate because brain tissue in adults does not show net growth: some material may be turned over, but the overall change in mass is negligible. Because ATP synthesis in the mitochondria is driven by the proton motive force across the inner membrane, the balance of mitochondrial protons determines the synthesis of ATP. Internal metabolites which are not exchanged are assumed to be balanced, which means that the fluxes producing and consuming the internal metabolite sum up to zero, i.e. flux balance is enforced. The flux distribution in the healthy brain was subsequently solved using the linear programming routine Linp from the package LIM [33] for the R programming environment.

### Estimating the flux distribution during disease

The flux distribution in the PD patients is subsequently estimated using the Lsei-FBA method, based on the changes in gene expression and the flux distribution in normal brain [3] calculated above. In brief, for each reaction, the average fold change from controls was computed for the expression of each gene associated with a biochemical reaction in the model (S2 Fig). The fold change for gene expression in the PD patients times the flux estimated for the associated biochemical reaction for the healthy brain yields the initial rough flux estimate for every reaction in the model.

In the next step, all flux estimations were refined based on flux balance in the model (S1 Fig). Under the assumption of absolute flux balance of the internal metabolites in the model and of zero backflux for the irreversible reactions, given in S1 Table, a cost function was minimized consisting of the sum of the squared deviations between final estimated flux and initial rough estimate of the flux as calculated above.

The equations of this problem of least squares with equalities (balanced fluxes) and inequalities (irreversible reactions) were solved using the least squares with equality and inequality conditions (lsei) method from the limSolve package [34]. This method, termed Lsei-FBA, has been described in detail in [3] and is a special case of quadratic programming.

### Statistical test for change of flux during disease

The difference in flux was calculated for n = 8 studies of gene expression in the substantia nigra, including two studies on dopaminergic neurons laser-dissected from that region. The significance of the difference in flux between normal controls and the eight predicted averages for whole SNc and dopaminergic neurons was tested using a one-sample t-test (p < 0.05). To control for multiple comparisons, the Family-wise Type 1 error (FWER) was calculated. Because the flux in a sequence of reactions that contains no side-branch is the same and these fluxes are therefore completely dependent, only one t-test was done per set of dependent fluxes, e.g. R_GLCt1r and R_HEX1 form one set, R_PGK, R_PGM, R_ENO and R_PYK form another set, etc.

Permutation analysis was performed to analyze the statistical significance of the predicted changes, taking the variability in gene expression in individual samples into account. For each dataset, 100,000 permutations were performed and the two-tailed p-values are calculated. A Fisher’s meta-analysis was performed that produced one p-value over all datasets. There are a total of 31 independent sets of reactions in the SNc and dopaminergic neurons datasets. Multiple testing is controlled by using the Family-wise Type 1 error: a Holm-Bonferroni correction with a *p* value = 0.05 was applied resulting in a threshold value of 0.0016.

## Results

### PD gene expression pattern across brain regions

Fold changes of mRNA expression of patients with PD against their healthy controls are visualized mapped on the reaction network in the substantia nigra and dopaminergic neurons in S2(A) Fig (SN datasets). Fold changes for the internal segment of the globus pallidus (GPi), putamen, frontal cortex, cerebellum, blood and lymphoblastoid cells are shown in S2(B) Fig (non-SN datasets). Downregulated genes are shown in green, upregulated genes in red.

The SN data for the expression in the glycolytic pathway shows mostly downregulation except for the hexokinases HK2 and HK3, phosphofructokinase PFKL and aldolase ALDOB genes. The solute carriers for glucose and lactate in the cell membrane tend to show upregulation. The expression changes in the pentose phosphate pathway (PPP) are small and mixed. Pathways in the mitochondria are generally downregulated, including the TCA cycle, oxidative phosphorylation and transfer of reducing equivalents across the mitochondrial membrane. However, the pyruvate dehydrogenase kinase PDK4, which participates in the regulation of pyruvate dehydrogenase activity, tends to show upregulation. Interestingly, the expression of mitochondrially encoded genes (mtDNA) in the electron transport chain (ETC) such as ND1, ND2, ND3, ND4, ND4L, ND5, ND6, CYTB, COX1, COX2, COX3, ATP6 and ATP8 are increased.

Outside the substantia nigra, transcription level changes are in general similar as in the SN datasets, with the GPi region (GSE20146) forming a clear exception. The GPi shows upregulation in most glycolytic genes while TCA cycle and oxidative phosphorylation genes are not downregulated and even show a tendency of slight upregulation (S2(B) Fig).

In general, changes in gene expression associated with biochemical reactions in the model were modest and tended to be in most cases in the same direction across the metabolic network, which makes the datasets well suited for flux quantification by the Lsei-FBA algorithm.

### Predicted flux distribution in the healthy brain

**Fig 2:**
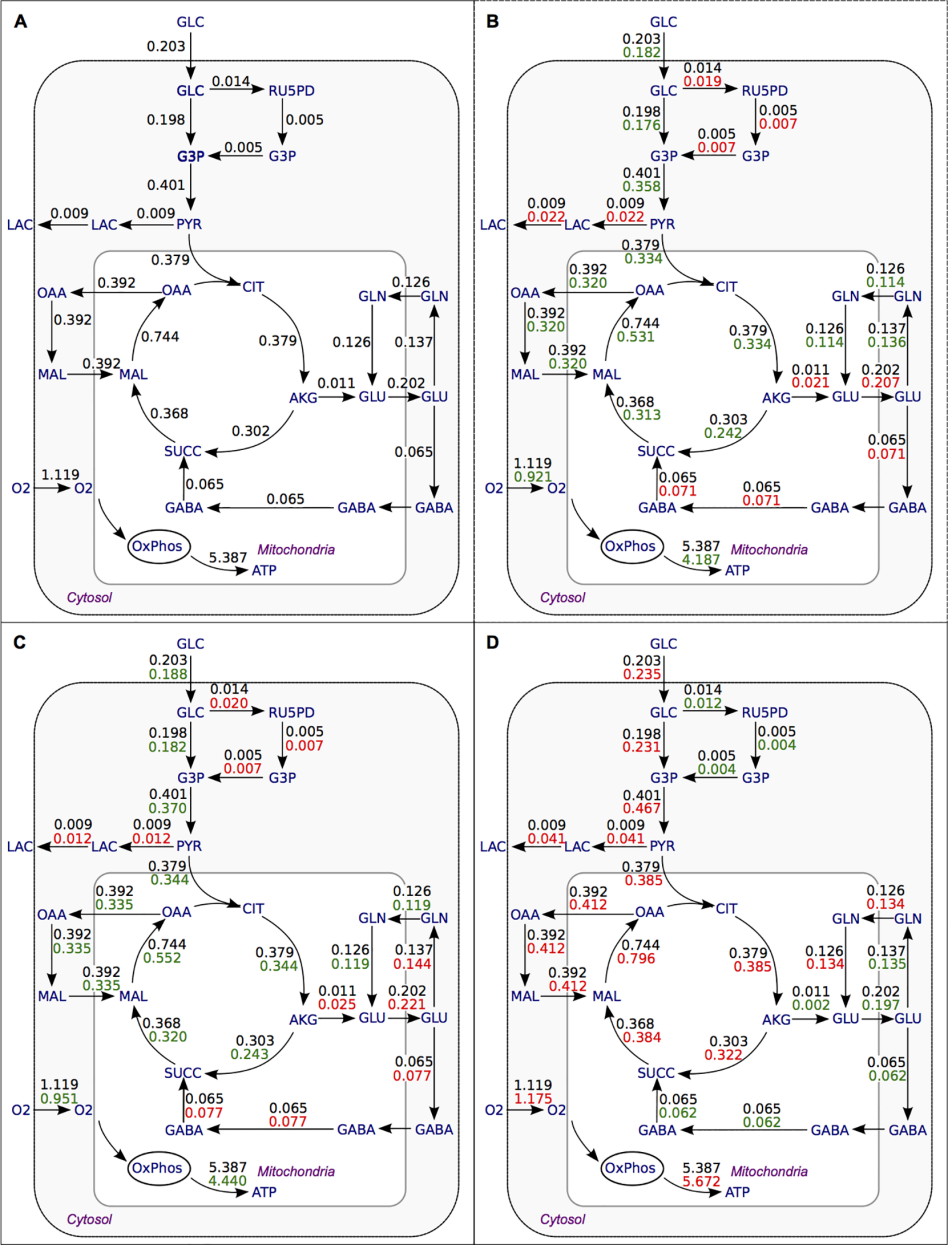
Flux distribution in healthy brain and during Parkinson’s disease. Flux distribution in healthy brain (A) and during Parkinson’s disease in the substantia nigra and dopaminergic neurons (B, average from eight data sets), averaged value for frontal cortex, BA9, putamen and cerebellum (C) and value for globus pallidus internus region (D) in μmol g (wet) brain^-1^ min^-1^. Black numbers, flux during normal condition; green numbers, flux decreased during PD and red numbers, increased from the normal condition. Note that for clarity not all separate biochemical steps are plotted: oxaloacetate is for instance first transaminated to aspartate before being transported across the mitochondrial membrane as part of the malate-aspartate shuttle. GLC, glucose; G3P, glyceraldehyde 3-phosphate; RU5PD, ribulose-5-phosphate; PYR, pyruvate; LAC, lactate; CIT, citrate; AKG, alpha-ketoglutarate; SUCC, succinate; MAL, malate; OAA, oxaloacetate; GLU, glutamate; GLN, glutamine, GABA, 4-aminobutanoate (synonym of gamma-aminobutyrate); O2, oxygen; OxPhos, oxidative phosphorylation. Flux values from GLC to RU5PD and from RU5PD to G3P represent 6-carbon units leaving the GLC pool rather than 3-carbon units entering the G3P pool.

Measurements show that 0.203 μmol g brain (wet)^-1^ min^-1^ of glucose is taken up in the normal brain of elderly people, and a small amount of lactate is excreted under baseline conditions [29]. Based on this metabolic input, the model analysis estimates that 5.39 μmol g brain (wet)^-1^ min^-1^ ATP is produced in the brain mitochondria. The predicted flux distribution is given in Fig 2A. The malate-aspartate shuttle transports reducing equivalents into the mitochondria. The glycerol phosphate shuttle is predicted to be inactive.

To investigate if the FBA yields unique values, we performed a Flux Variability Analysis (FVA) [35], to estimate the feasible minimum and maximum of all fluxes. The FVA proved that the calculated fluxes represent indeed a unique solution for this model (data not shown).

### Predicted flux distribution during Parkinson’s disease

We now predict changes in the metabolic flux distribution from the changes in gene expression data between the normal brain and PD. In most cases, fluxes are decreased from control for the substantia nigra and dopaminergic neuron gene expression datasets. A full list of flux predictions for the substantia nigra and its dopaminergic neurons is given in S4 Table. The glycolytic flux is predicted to be reduced by 10% on average during PD, while flux into the TCA cycle decreases by 12% and 6% of pyruvate influx is used to produce lactate (Fig 2B). The malate-aspartate shuttle carrying reducing equivalents into the mitochondria is reduced by 18%. In addition, flux in the pentose phosphate pathway increases and the glycerol phosphate shuttle becomes slightly active. Total export of ATP from the mitochondria decreases by 20% to 4.307 μmol g wet brain^-1^ min^-1^. In PD, on average, the GABA shunt is increased slightly (about 10%), partially compensating for the measured reduction in alpha ketoglutarate dehydrogenase (AKGDH) expression, whose flux is reduced by 20%. It is striking that the modest decrease in glucose uptake leads to an appreciably larger relative decrease in ATP production.

Flux changes in the frontal cortex, BA9, putamen and cerebellum during PD follow the same pattern as in the SN although with slightly smaller changes (Fig 2C and S5 Table). The change in fluxes in the globus pallidus internus is quite different from the substantia nigra and all other regions. In the GPi, increased flux from the normal condition is predicted in most of the pathways: glycolysis increased by 16%, lactate production is 9% of glycolytic flux, malate-aspartate shuttle is 5% higher, TCA cycle and OxPhos are on average 5% higher (Fig 2D). An increase in ATP production to 5.35 μmol g wet brain^-1^ min^-1^ through oxidative phosphorylation is predicted, utilizing 1.17 μmol g wet brain^-1^ min^-1^ of oxygen. In this case the AKGDH flux is not reduced as in other brain regions, but slightly increased while the GABA shunt, AKGDH’s potential bypass, is slightly reduced.

Permutation analysis showed that the change in metabolic fluxes predicted from gene expression changes during Parkinson’s disease was highly significant in the metabolic network: taken over all studies which showed substantial variation, all reaction rates were significantly affected during Parkinson’s disease, except CO_2_ production and glutamine synthetase (S4 Table).

For the solution in the diseased brain obtained with the ‘Least-squares with equalities and inequalities’ algorithm, we proved that the solution was unique in the following way: we systematically tested for each reaction whether a different solution with equally low cost function existed if the flux in this reaction was forced to be slightly lower or higher than the original solution. To this end we first added an upper bound for the reaction which was tested to the Lsei problem, setting this to a value that was lower than the flux value in the original solution by a very small number (0.00001 times the flux value, e.g. 1.93846 x 10^−6^ for a predicted flux value of 0.193846), and then calculated the minimal sum of squares of these reaction fluxes with this additional constraint. For the same reaction, we separately also added a lower bound to the Lsei which was higher than the flux in the original solution by a very small number, again testing whether an equally low sum of squares was found as in the original solution. In this way we proved that in each reaction in all datasets, the minimal sum of squares is higher than the original calculated sum of squares if any reaction flux is forced to be displaced from the original solution by even a very small amount, thus proving that the solution is unique.

## Discussion

### Comparison of computational predictions and cerebral metabolic measurements

To test the Lsei-FBA method to calculate changes in metabolism from changes in expression of metabolic genes, we compare changes predicted with direct measurements that were possible in relatively large brain regions. Measurements of cerebral metabolism in PD by positron emission tomography (PET) have recently been meta-analyzed [12,36]: in 11 out of the 14 studies, 2–32% decreases in cerebral glucose consumption were reported, although in only four of these cases the change was reported to be significant. In only two of the meta-analyzed studies there was a very small (2–4%) and non-significant increase. From the gene expression changes in cortical areas analyzed in the present study (GSE8397 and GSE20168; see S5 Table) we predict a decrease in glucose consumption of about 11%, which is of the same order as the 8.5% average decrease seen in the meta-analysis of the PET measurements.

In the meta-analysis, the decrease in oxygen consumption in three PD study groups measured by PET ranged from 6–34% (average 19% decrease). From the gene expression changes in the two cortical areas (S5 Table), we computationally predict a decrease of 17.5 and 22% in oxygen consumption. Our predictions for changes in glucose and oxygen consumption for the cortical areas agree with direction and size of change in the PET measurements in PD patients. Our computational predictions are compatible with the conclusion from the meta-analysis of PET measurements that in PD there is cortical hypometabolism [12,36]. A summary of the comparison of our prediction based on the Lsei-FBA algorithm and experimental observation is given in Table 1. Given the correspondence between flux changes predicted by the Lsei-FBA algorithm and experimental results compiled in Table 1, it appears that the direction and order of magnitude of flux changes during Parkinson’s disease is predicted correctly by the algorithm. Thus, the hypothesis underlying the Lsei-FBA algorithm appears to give useful results, at least for some biochemical pathways. The results suggest that the predictions are not fully accurate, but direction and order of magnitude of the predictions correspond with measurements. Both algorithm predictions and experimental suggest that changes in metabolism during Alzheimer disease [3] are much larger than during Parkinson’s disease (present study). Flux predictions for pathways not given in Table 1 have not yet been corroborated, but are reported as potential changes in metabolism that may be of interest and merit further investigation.

**Table 1:** Comparison between the predictions of the Lsei-FBA algorithm based on gene expression changes and experimental observations.

Quantity	Prediction by algorithm	Experimental observation	References
Glucose uptake in cortex	11% decrease during PD	8.5% decrease (range 2 to 32%)	Meta-analysis [12,36]
Oxygen uptake in cortex	17.5 and 22% decrease in two cortical areas	19% decrease (range 6–34%)	Meta-analysis [12,36]
Lactate levels	2 ½ fold increase in lactate production	Increased lactate concentration measured by NMR spectroscopy	[13,37]
NADPH production in pentose phosphate pathway	1 1/3 fold increase in NADPH production	In vitro assay: ~1.4 fold increase in NADPH production	[38]
α-ketoglutarate dehydrogenase	Decreased flux: e.g. in cerebellum by 23%	Decreased enzyme activity (by 50% in cerebellum) and immunohistochemical reduction in substantia nigra	[39,40]
Activity brain region	Globus pallidus internus: increased metabolic activity, in contrast to other regions	Globus pallidus internus: increased neuronal firing rates	[41]

The spatial resolution of PET measurements is characterized by a Full Width at Half Maximum of at least 5 mm, which in practice is often even considerably larger [12]. In contrast, gene expression measurements were even feasible for laser-excised cells which made computational predictions specifically for dopaminergic neurons possible. Further, in addition to glucose and oxygen uptake, our computational method describes the metabolic pattern in the entire network and therefore potentially has a high ‘biochemical resolution’ while with PET uptakes of single metabolites are measured. The computational predictions by the present algorithm are for instance (1) that the relative decrease in ATP synthesis is larger than the decrease in glucose uptake, (2) that the flux in the middle of the TCA cycle is more reduced than at the entry point of acetyl CoA, (3) limited metabolic rerouting around downregulated enzymes, (4) shifts in redox shuttles and (5) emergence of lactate production in at least part of the study groups. The last prediction is in agreement with NMR measurements (see below).

### Predicted metabolic fluxes during Parkinson’s disease

ATP synthesis is driven by protons which are pumped by the ETC complexes from mitochondrial matrix to cytosol and flow back through ATP synthase. In our network model, protons in the mitochondrial matrix are balanced. Our computational analysis predicts that the proton fluxes through all ETC complexes and ATP synthase in the SNc during PD are reduced by the same proportion (average 18%) relative to the healthy brain. As a result, the predicted uptake of oxygen in the brain and the ratio of oxygen to glucose uptake are reduced.

Our computational analysis predicts that the reduced pyruvate flux into the mitochondria is associated with production of lactate, accounting for about 10% of pyruvate in the GPi region and for about 6% in the substantia nigra and other brain regions. Increase in cerebral lactate in PD has indeed been measured using magnetic resonance spectroscopy in various parts of the brain [13,37] (Table 1).

The pentose phosphate pathway (PPP) is an important branch of glucose metabolism that supplies NADPH, an important cofactor for antioxidant defense of brain cells by glutathione (GSH) redox cycling. PPP flux in the brain of traumatic brain injury (TBI) patients increases by 19.6% relative to glucose uptake [30]. Dunn et al. [38] suggested disruption in glucose metabolism through PPP dysregulation as an important mechanism in the pathogenesis of Parkinson’s disease. However, they reported somewhat increased NADPH production by the PPP, assayed in vitro in brain homogenates of moderate to severe Parkinson disease patients, in agreement with the prediction (Fig 2) for in vivo metabolism by the present model [38] (Table 1).

### Rerouting of pathways

In the GABA shunt pathway, the flux of alpha-ketoglutarate to succinate in the TCA cycle via alpha-ketoglutarate dehydrogenase (AKGDm) and succinate-CoA ligase (SUCOAS1m) is rerouted through decarboxylation of glutamate to GABA via glutamate decarboxylase (GLUDC) in the cytosol, and subsequently to succinate via GABA transaminase (ABTArm) and succinate semialdehyde dehydrogenase (SSALxm) in the mitochondria (S1 Fig). The GABA shunt is active in GABAergic neurons [42], providing a mechanism for synthesis of GABA which is an inhibitory neurotransmitter. The GABA shunt in general accounts for less than half of the total TCA cycle flux in GABAergic neurons [42,43]. GABAergic neurons account for about 18% of total neuronal glucose oxidation [31]. The GABA shunt flux is present in glutamatergic and cholinergic neurons, although it is small there [44].

In PD, a marked reduction in alpha-ketoglutarate dehydrogenase (AKGDm) complex by immunostaining has been reported in the substantia nigra of PD patients [39]. Gene expression data associated with AKGDm also show downregulation in PD patients (S2 Fig). Consistent with this reduced activity, the computational analysis also predicts lower flux through AKGDm. This reduction can in principle be compensated by rerouting of alpha-ketoglutarate through the GABA shunt. For Alzheimer’s disease (AD), Lewis et al. [44] applied a metabolic model and inferred that the about 50% reduced AKGDm activity measured for AD is compensated by increases in GABA shunt flux (Table 1). We confirmed this prediction based on the Lsei-FBA analysis of gene expression changes in an AD data set [3]. However, in the present study the upregulation of flux in the GABA shunt pathway during PD was predicted to be much smaller than for AD.

### Flux in globus pallidus internus is increased

The present flux analysis predicted total cellular ATP production in the GPi region of the brain to be higher during PD, accompanied by increased fluxes in all pathways (Fig 2D). This may be associated with the role of GPi in the neural circuits that regulate human movement. In PD, loss of dopaminergic neurons in the striatum causes hyperactivation of the subthalamic nucleus (STN) and GPi, leading to increased neuronal firing rates in the GPi [41] and disturbed regulation of motor neurons [45,46]. This theory has been the basis of deep brain stimulation (DBS) treatment in PD patients targeting the GPi and STN region [45]. This explains the correspondence between increased in GPi activity and decrease activity in the SNc and putamen. There is a striking correspondence between the direction of the change in predicted metabolic activity and the reported connectivity of these brain regions. A remarkable detail is that in spite of increased oxygen uptake, lactate efflux in the GPi is predicted to be increased.

### Limitations and prospects of the study

By computational analysis we predicted changes in metabolic fluxes in small regions in the brain, such as the substantia nigra. In relatively large cortical regions the metabolic rates for glucose and oxygen were measured with PET, agreeing with our computational predictions. Metabolic changes in small regions such as the substantia nigra, and in particular in dopaminergic neurons in this region, could not a priori be assumed to be the same as changes determined in larger regions which are accessible to experimental flux measurements with low spatial resolution. However, the present computational analysis predicts changes in the SN that are similar to other brain regions. Also the results for laser-captured dopaminergic neurons are similar to the whole SN and most other brain regions. In contrast, one particular brain region, the GPi, shows different metabolic changes than other brain regions, including the SN which usually is most prominently affected by PD. Our computational prediction therefore suggests that during PD, metabolism is decreased similarly in most brain regions. However, the GPi represents a small region where metabolism is increased in parallel with increased neuronal activity.

The gene expression measurements used for predictions in flux changes in the diseased brain in this study are taken from tissue samples from various part of the brain including the substantia nigra, BA9, cerebellum, putamen, frontal cortex, globus pallidus internus as well as from blood and lymphoblastoid, and we could see hints of connectivity between brain regions as discussed above. Connectivity between regions could in principle be investigated further, however this may require gene expression measurements and prediction of metabolism in the regions under a variety of conditions which may not be practical given the invasive nature of the experimental protocols. Non-invasive imaging methods enabling repeated measurements in the same subject are therefore better suited for studying connectivity between brain regions. However, the present methodology is useful in predicting metabolism in many biochemical reactions in brain regions of patients in comparison with healthy controls.

The predicted changes in metabolism are averages for the region sampled based on gene expression changes measured for the sample as a whole. There are several distinct cell types inside these regions. The disease may have progressed much more in some of the cells than in others, and damage may even be heterogeneous for cells of the same type. The changes in metabolic fluxes may therefore be larger in a subset of the cells than in the tissue as a whole. Because neurons and glia are lumped in the most of the mRNA expression measurements, we also used a model which lumps metabolism of neurons and glia. Models of brain metabolism with separate compartments for neurons and glial cells exist [44,47], but have no added value in this case because the available gene expression measurements reflect a weighted average of cell types. For the present analysis a lumped model was therefore used with biochemical reactions not compartmentalized in distinct cell types. The use of a metabolic model with one compartment for tissues which actually contain several cell types means that limitation of metabolism by exchange processes between the cells is assumed to be negligible. The correspondence found between metabolic rate measurements and computational predictions for cortical regions, see above, is compatible with this assumption.

The Lsei approach can be used to compare patients with different disease states to assess how different brain regions change specifically and how sensitive different brain regions are to disease states. However, in the present study on PD, datasets were not available with sufficient specification and differentiation of the disease states. We are presently analysing this method for Alzheimer’s disease datasets, which include expression data in incipient, moderate and severe stage where the expression changes are clearly larger in severe cases. Predicted flux changes in severe cases tend to be larger especially in the fluxes in the glycolytic pathway, ATP synthesis and oxygen uptake. The sensitivity can be affected by the specific region where the gene expression measurement is taken from. For example, changes in excised neurons (dopaminergic neurons) may be higher than in the whole tissue.

Among the SN expression datasets included in the study by Zheng et al. [11], the dataset from Cantuti-Castelvetri et al. [23] (GEO accession GSE24378) differs from the rest by displaying overexpression during Parkinson’s disease in most of the genes in the metabolic pathways. As suggested by [11], this may be caused by the use of the non-standard X3P microarray chip, which differs from the rest of the platforms used. For this reason this data set was not included in the final analysis of the present study. Our analysis on the GSE24378 data set indeed predicted that most metabolic fluxes are upregulated (see S4 Table), which differs from the results for all other SN data sets.

The present prediction is based on gene expression changes. Regulation of translation of mRNAs in proteins and breakdown and posttranslational modification and allosteric regulation of enzymes in the metabolic network may modify the relation between mRNA expression and flux. The relation between changes in gene expression and metabolic fluxes was investigated for glycolysis in yeast [1]. Only a fraction of the enzymes involved in yeast glycolysis showed clear changes in gene expression in the same direction as the change in flux carried by that particular enzyme. This is confirmed by a recent study in yeast, which showed that enzyme protein levels explained only a relatively modest part, about a quarter, of variation in flux through the reaction which they catalyzed [48]. However, the Lsei-FBA algorithm assumption applies to the whole network level. In our study, the changes in gene expression in metabolic pathways in PD (S2 Fig) appeared to be more consistent and uniform than in the studies on yeast glycolysis. This may explain why the computational predictions based on gene expression changes in the present study agree with the changes in metabolic rate measured by PET (see above).

A recent approach presents flux prediction based on absolute gene expression data on a large scale yeast network [28]. The latter method is able to meaningfully predict flux compared to exo-metabolome measurements. The approach by Lee et al. [28] in yeast and our present approach have a common assumption that metabolic fluxes tend to be related to gene expression without assuming a rigid relation at the level of each individual reaction. Both studies suggest that it is useful to take the metabolic network connectivity into account to estimate an overall effect of gene expression on the metabolic flux.

Several other algorithms exist to predict metabolic fluxes from gene expression data. These algorithms, such as iMAT [4], GIMME [49], GX-FBA [50], E-Flux [51], Lee-12 [28], RELATCH [52] have recently been extensively reviewed and benchmarked on yeast and E. coli data [2]. In the original publication on Lsei-FBA, the algorithms tested by Machado were tested on gene expression data for brain tissue [3] and appeared to perform better for this application than the algorithms benchmarked by Machado et al. [2]. The characteristics of Lsei-FBA in comparison with these other algorithms have already been extensively discussed by Gavai et al. [3].

Our approach has a limitation which is specific to brain tissue: a fraction of the enzymes which are formed from the measured messenger RNAs are transported over relatively long distances to catalyze metabolism in axonal terminals. Many dopaminergic neurons in the SNc receive for instance input via GABAergic synapses from relatively distant GABAergic neuronal cell bodies [53]. The predicted metabolic changes therefore apply to the cells whose gene expression levels are measured, which includes distant nerve terminals of those cells, but does not apply to metabolic changes in nerve terminals from distant neuronal cell bodies that extend into the region where mRNAs are sampled. This means that metabolic changes predicted from gene expression changes on the one hand, and directly measured in the same region on the other hand, may diverge to a certain extent.

The comparison of flux changes predicted by the Lsei-FBA algorithm and experimental data (see Table 1) suggests the usefulness of the hypothesis of parallel changes in fluxes in biochemical pathways and gene expression when evaluated at the level of a whole metabolic network. This is perhaps surprising given that the flux-gene expression relation at the level of individual enzymes and biochemical reactions is relatively weak. Nevertheless, the fact that the predictions work, at least for some biochemical pathways, suggests that gene expression levels are meaningfully related to metabolic system function, even during pathological processes, and underscores the often used assumption that gene expression levels can be used as indicator of changes in biological pathway activity.

Presently, perhaps the most important limitation for the application of the Lsei-FBA algorithm is the limited possibility to compare its predictions with direct metabolic measurements in patients and controls in vivo. Comparison with measured oxygen and glucose uptake, lactate levels, NADPH production in the PPP and with physiological activity in the GPi region was feasible (Table 1), but other predictions of the algorithm await confirmation by measurements. The other side of the coin is that the model predictions allow insight into potential changes in intracellular metabolism that presently escape measurement capabilities.

## Conclusions

This paper describes application of a recent method to predict changes in metabolic fluxes based on changes in gene expression in patient material. The hypothesis underlying the Lsei-FBA algorithm. that fluxes in biochemical pathways change in parallel to gene expression if analyzed at the metabolic network level, appears to give useful predictions, at least for some biochemical pathways. From gene expression changes during Parkinson’s disease, metabolic fluxes through central carbon metabolism are predicted to be reduced in the substantia nigra and other brain regions including frontal cortex, cerebellum and putamen. A striking result is that the predicted relative changes in ATP synthesis are larger than the changes in glucose uptake. We also predicted increase of lactate production and shifts in redox shuttles. Reduced metabolism via alpha ketoglutarate dehydrogenase in the middle of the TCA cycle is less compensated via the GABA shunt than is the case in Alzheimer’s disease. In contrast to the decreases in metabolism in substantia nigra and most other brain regions, the globus pallidus internus part of the brain is predicted to show increased metabolic flux compared to normal controls.

## Supporting information

S1 Table List of the reactions in the model. (XLS)

S2 Table List of the metabolites in the model. (XLS)

S3 Table Summary of all datasets used in this study. (XLS)

S4 Table Results for flux prediction for substantia nigra and dopaminergic neuron datasets. (XLSX)

S5 Table Results for flux prediction for other tissues. (XLS)

S1 Fig Reconstructed metabolic reaction network. (PDF)

S2 Fig Visualization of fold changes in mRNA expressions are mapped on the metabolic pathway model. (PDF)
