# Supplementary material for: Computational prediction of changes in brain metabolic fluxes during Parkinson’s disease from mRNA expression
Source: PLoS One. 2018 Sep 12;13(9):e0203687. doi: 10.1371/journal.pone.0203687 (PMC6135490; doi:10.1371/journal.pone.0203687)
Supplement: S1 Fig — (PDF) [file pone.0203687.s006.pdf]

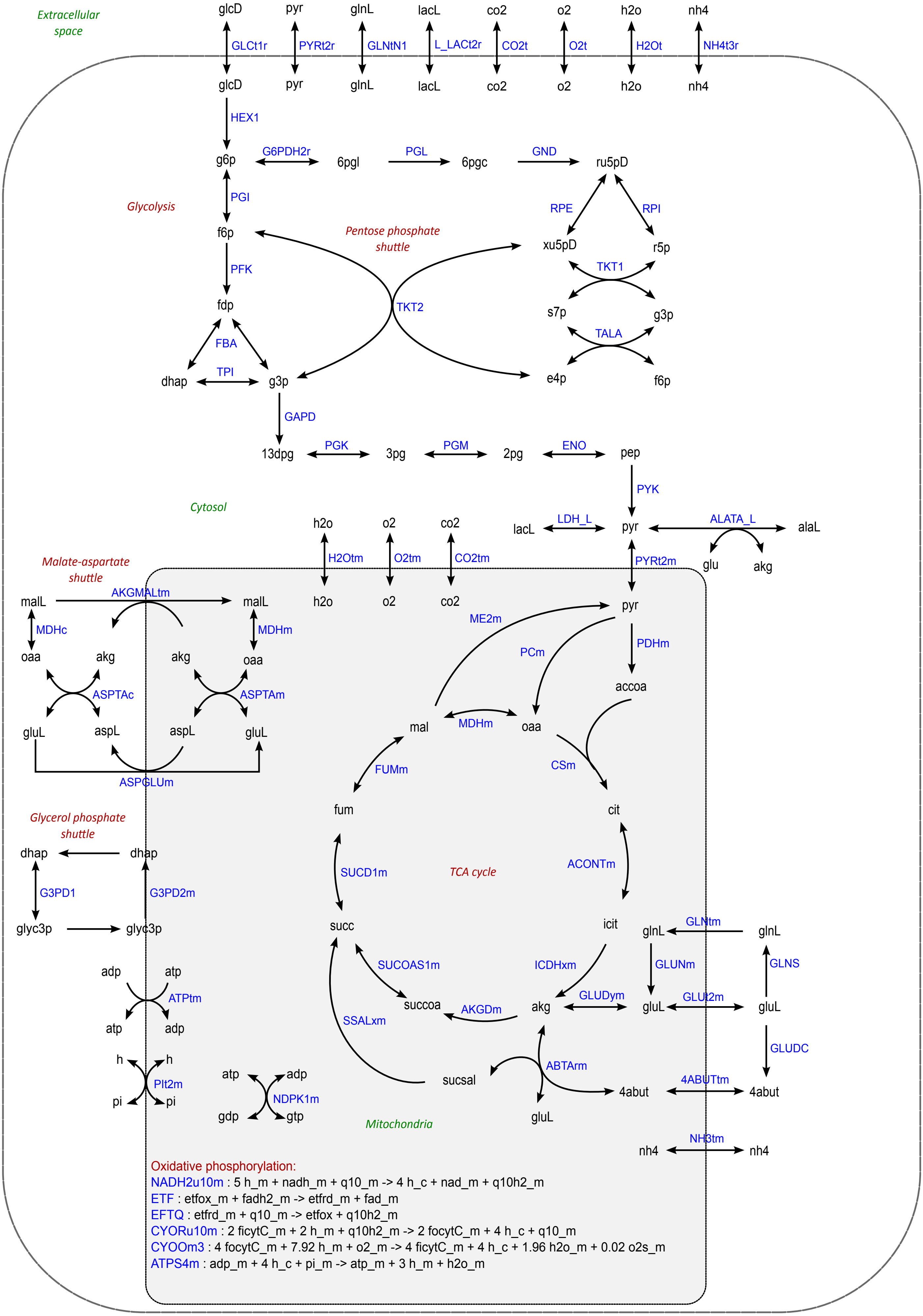
Extracellular space
glcD
pyr
glnL
lacL
co2
o2
h2o
nh4
GLCt1r
PYRt2r
GLNtN1
L_LACt2r
CO2t
O2t
H2Ot
NH4t3r
glcD
pyr
glnL
lacL
co2
o2
h2o
nh4
HEX1
g6p
G6PDH2r
6pgl
PGL
6pgc
GND
ru5pD
PGI
Glycolysis
RPE
RPI
f6p
Pentose phosphate shuttle
xu5pD
r5p
TKT1
PFK
fdp
TKT2
s7p
g3p
FBA
TALA
TPI
dhap
g3p
e4p
f6p
GAPD
13dpg
PGK
3pg
PGM
2pg
ENO
pep
PYK
Cytosol
h2o
o2
co2
lacL
LDH_L
pyr
ALATA_L
alaL
H2Otm
O2tm
CO2tm
PYRt2m
glu
akg
Malate-aspartate shuttle
AKGMALtm
malL
malL
h2o
o2
co2
pyr
MDHc
MDHm
ME2m
PDHm
oaa
akg
akg
oaa
PCm
accoa
ASPTAc
ASPTAm
gluL
aspL
aspL
gluL
mal
MDHm
oaa
FUMm
CSm
ASPGLUm
Glycerol phosphate shuttle
fum
cit
dhap
dhap
G3PD1
G3PD2m
SUCD1m
TCA cycle
ACONTm
glyc3p
glyc3p
succ
icit
glnL
GLNtm
glnL
SUCOAS1m
ICDHxm
GLUNm
GLNS
adp
atp
ATPtm
atp
adp
succoa
AKGDm
akg
GLUDym
gluL
GLUt2m
gluL
SSALxm
GLUDC
h
h
PIt2m
pi
pi
atp
adp
NDPK1m
gdp
gtp
sucsal
ABTArm
4abut
4ABUTtm
4abut
Mitochondria
gluL
nh4
NH3tm
nh4
Oxidative phosphorylation:
NADH2u10m : 5 h_m + nadh_m + q10_m -> 4 h_c + nad_m + q10h2_m
ETF : etfox_m + fadh2_m -> etfrd_m + fad_m
EFTQ : etfrd_m + q10_m -> etfox + q10h2_m
CYORu10m : 2 ficytC_m + 2 h_m + q10h2_m -> 2 focytC_m + 4 h_c + q10_m
CYOOm3 : 4 focytC_m + 7.92 h_m + o2_m -> 4 ficytC_m + 4 h_c + 1.96 h2o_m + 0.02 o2s_m
ATPS4m : adp_m + 4 h_c + pi_m -> atp_m + 3 h_m + h2o_m

Supplementary Figure S3: Reconstructed metabolic reaction network.
Reactions considered in this model are given in Supplementary Table S1. Reaction names are based on the BiGG database.
