# Supplementary material for: Computational prediction of changes in brain metabolic fluxes during Parkinson’s disease from mRNA expression
Source: PLoS One. 2018 Sep 12;13(9):e0203687. doi: 10.1371/journal.pone.0203687 (PMC6135490; doi:10.1371/journal.pone.0203687)
Supplement: S2 Fig — (PDF) [file pone.0203687.s007.pdf]

A) SNc data

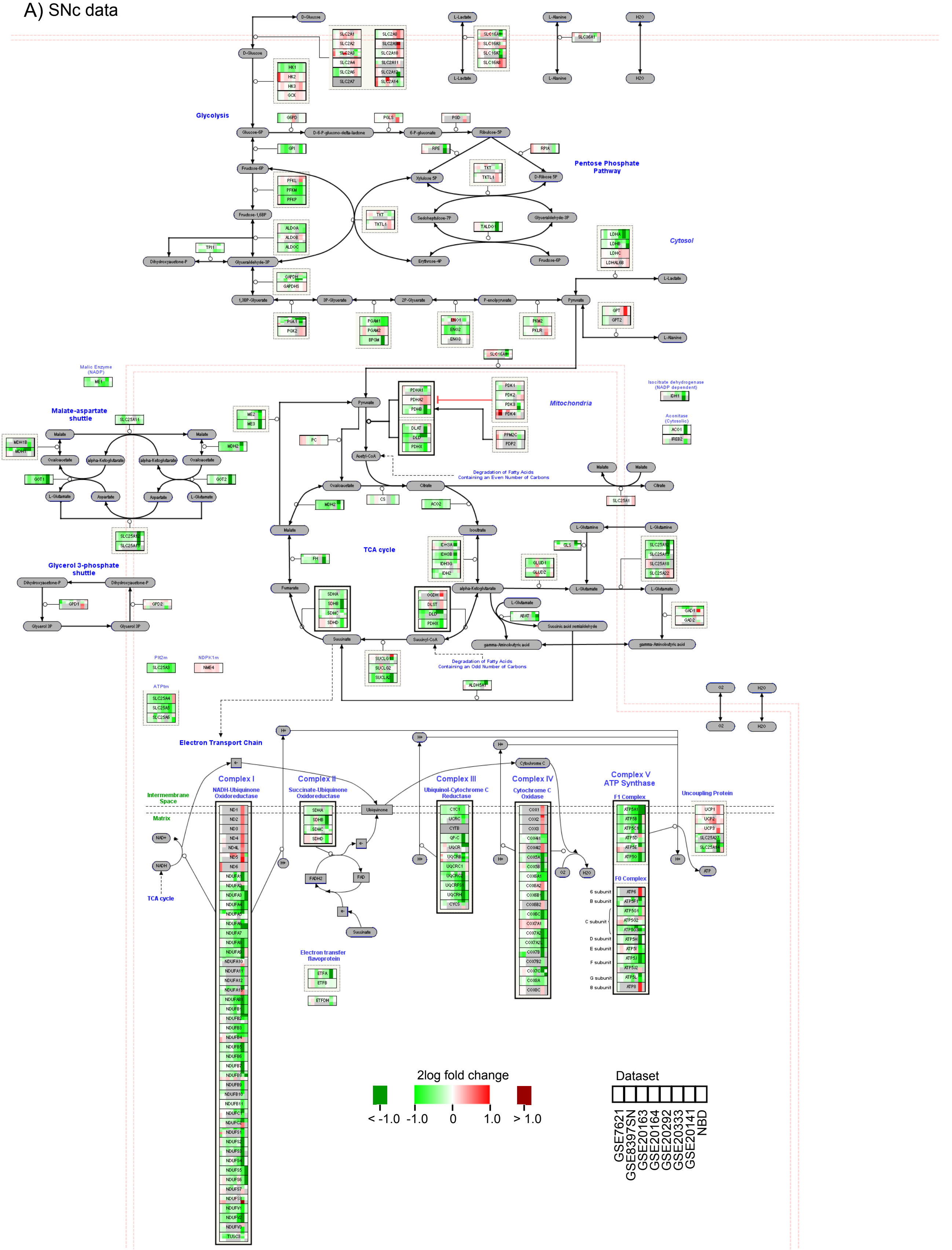

D-Glucose
SLC2A1
SLC2A2
SLC2A3
SLC2A4
SLC2A6
SLC2A7
SLC2A8
SLC2A9
SLC2A10
SLC2A11
SLC2A12
SLC2A14
L-Lactate
SLC16A1
SLC16A3
SLC16A7
SLC16A8
L-Alanine
SLC38A1
H2O
HK1
HK2
HK3
GCK
Glycolysis
G6PD
PGLS
PGD
Glucose-6P
D-6-P-glucono-delta-lactone
6-P-gluconate
Ribulose-5P
GPI
RPE
RPIA
Fructose-6P
Xylulose 5P
TKT
TKTL1
D-Ribose 5P
Pentose Phosphate Pathway
PFKL
PFKM
PFKP
Fructose-1,6BP
Sedoheptulose-7P
Glyceraldehyde-3P
TALDO1
ALDOA
ALDOB
ALDOC
TPI1
Dihydroxyacetone-P
Glyceraldehyde-3P
Erythrose-4P
Fructose-6P
LDHA
LDHB
LDHC
LDHAL6B
Cytosol
GAPDH
GAPDHS
1,3BP-Glycerate
3P-Glycerate
2P-Glycerate
P-enolpyruvate
Pyruvate
L-Lactate
GPT
GPT2
L-Alanine
PGK1
PGK2
PGAM1
PGAM2
BPGM
ENO1
ENO2
ENO3
PKM2
PKLR
SLC16A1
Malic Enzyme (NADP)
ME1
Isocitrate dehydrogenase (NADP dependent)
IDH1
Aconitase (Cytosolic)
ACO1
IREB2
Malate-aspartate shuttle
SLC25A11
Malate
MDH1B
MDH1
MDH2
Oxaloacetate
alpha-Ketoglutarate
GOT1
GOT2
L-Glutamate
Aspartate
SLC25A12
SLC25A13
ME2
ME3
Pyruvate
PDHA1
PDHA2
PDHB
DLAT
DLD
PDHX
PDK1
PDK2
PDK3
PDK4
PPM2C
PDP2
Mitochondria
PC
Acetyl-CoA
Degradation of Fatty Acids Containing an Even Number of Carbons
Malate
Citrate
SLC25A1
Oxaloacetate
CS
ACO2
MDH2
Malate
Isocitrate
TCA cycle
L-Glutamine
GLS
SLC25A12
SLC25A13
SLC25A18
SLC25A22
IDH3A
IDH3B
IDH3G
IDH2
GLUD1
GLUD2
FH
Glycerol 3-phosphate shuttle
Dihydroxyacetone-P
GPD1
GPD2
Glycerol 3P
Fumarate
SDHA
SDHB
SDHC
SDHD
OGDH
DLST
DLD
PDHX
alpha-Ketoglutarate
L-Glutamate
ABAT
Succinic acid semialdehyde
GAD1
GAD2
Succinate
Succinyl-CoA
gamma-Aminobutyric acid
SUCLG1
SUCLG2
SUCLA2
Degradation of Fatty Acids Containing an Odd Number of Carbons
ALDH5A1
Pit2m
SLC25A3
NDPK1m
NME4
ATPtm
SLC25A4
SLC25A5
SLC25A6
O2
H2O
Electron Transport Chain
H+
e-
Cytochrome C
Complex I
NADH-Ubiquinone Oxidoreductase
Complex II
Succinate-Ubiquinone Oxidoreductase
Complex III
Ubiquinol-Cytochrome C Reductase
Complex IV
Cytochrome C Oxidase
Complex V
ATP Synthase
F1 Complex
Uncoupling Protein
Intermembrane Space
Matrix
Ubiquinone
NAD+
NADH
TCA cycle
FADH2
FAD
Succinate
ND1
ND2
ND3
ND4
ND4L
ND5
ND6
NDUFA1
NDUFA2
NDUFA3
NDUFA4
NDUFA5
NDUFA6
NDUFA7
NDUFA8
NDUFA9
NDUFA10
NDUFA11
NDUFA12
NDUFA13
NDUFAB1
NDUFB1
NDUFB2
NDUFB3
NDUFB4
NDUFB5
NDUFB6
NDUFB7
NDUFB8
NDUFB9
NDUFB10
NDUFB11
NDUFC1
NDUFC2
NDUFS1
NDUFS2
NDUFS3
NDUFS4
NDUFS5
NDUFS6
NDUFS7
NDUFS8
NDUFV1
NDUFV2
NDUFV3
TUSC3
UQCR
CYC1
UCRC
CYTB
QP-C
UQCR
UQCRB
UQCRC1
UQCRC2
UQCRFS1
UQCRH
CYCS
COX1
COX2
COX3
COX4I1
COX4I2
COX5A
COX5B
COX6A1
COX6A2
COX6B1
COX6B2
COX6C
COX7A1
COX7A2
COX7A2L
COX7B
COX7B2
COX7C
COX8A
COX8C
O2
H2O
ATP5A1
ATP5B
ATP5C1
ATP5D
ATP5E
ATP5O
F0 Complex
6 subunit
B subunit
C subunit
D subunit
E subunit
F subunit
G subunit
8 subunit
ATP6
ATP5F1
ATP5G1
ATP5G2
ATP5G3
ATP5H
ATP5I
ATP5J
ATP5J2
ATP5L
ATP8
UCP1
UCP2
UCP3
SLC25A27
SLC25A14
ATP
Electron transfer flavoprotein
ETFA
ETFB
ETFDH
2log fold change
< -1.0
-1.0
0
1.0
> 1.0
Dataset
GSE7621
GSE8397SN
GSE20163
GSE20164
GSE20292
GSE20333
GSE20141
NBD

B) Other tissues

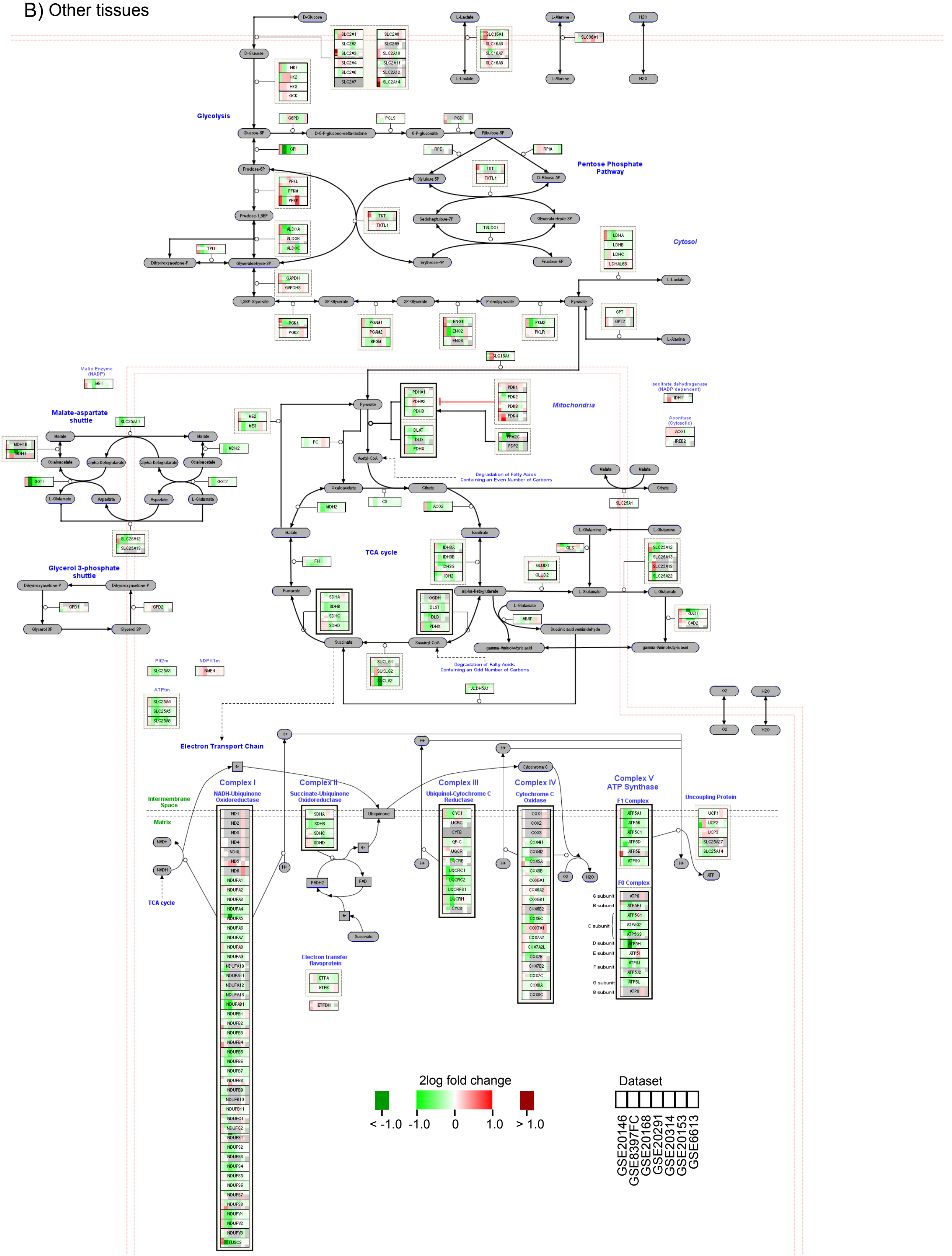

Supplementary Figure S6: Visualization of fold changes in mRNA expressions are mapped on the metabolic pathway model.

Each rectangular box represents a gene for a protein involved in a biochemical reaction. Stacks of boxes represent isoforms (several different genes with the same function: enclosed in boxes with dashed borders) or complexes (several gene products together form a large structure: enclosed in boxes with continuous borders). These colored boxes connect the metabolites (rounded boxes with grey filling) in a pathway. The rectangular boxes representing enzymes are filled from left to right with small colored squares with the color coding indicating the fold change measured in the patient groups as shown in the legend. (A) shows the substantia nigra (first 6 from the left) and the remaining two on the right are dopaminergic neurons. (B) shows the other brain regions, from left to right: globus pallidus internus, frontal cortex, Brodmann area 9, putamen, cerebellum, lymphoblastoid cells and blood. Expression colors: green, downregulation; red, upregulation; grey, no expression data available for the associated probesets.
